# Structure–function association of the cerebellar motor network is altered in isolated cervical dystonia

**DOI:** 10.1007/s00415-025-13186-x

**Published:** 2025-06-03

**Authors:** Kai Grimm, Hanna Braaß, Fatemeh Sadeghi, Mathias Gelderblom, Robert Schulz, Simone Zittel

**Affiliations:** 1 Department of Neurology, University Medical Center Hamburg-Eppendorf, Martinistr. 52, 20246 Hamburg, Germany; 2 Institute of Systems Neuroscience, University Medical Center Hamburg-Eppendorf, Hamburg, Germany

**Keywords:** Cervical dystonia, Cerebellum, Magnetic Resonance Imaging, Transcranial Magnetic Stimulation, Transcranial Direct Current Stimulation

## Abstract

**Background:**

Cervical dystonia (CD) has been recognized as a disorder of the brain’s sensorimotor network. Within this malfunctioning network, the cerebellum plays an important role that needs to be further characterized.

**Methods:**

To investigate the structural connectivity of the dentato-rubro-thalamic tract (DRTT), probabilistic tractography was performed in 18 CD patients and 18 matched healthy control (HC) subjects. Connectivity was quantified with fractional anisotropy (FA). Thirteen subjects in each group also participated in a neurophysiological double-blind experiment to investigate the effect of cathodal and sham cerebellar transcranial direct current stimulation (ctDCS) on sensorimotor associative plasticity, as evoked by paired associative stimulation (PAS). The association of FA of the DRTT and neurophysiological parameters was studied with linear models.

**Results:**

The FA of the DRTT was not different between the groups and not related to motor symptom severity in CD patients. In the HC group, there was a significant association between the structural connectivity of the DRTT and the effect that cathodal ctDCS had on the PAS effect. This association was not found in CD patients.

**Conclusions:**

The microstructural state of the DRTT is a potential biomarker for the efficacy of ctDCS in HC. The lack of this structure–function association in patients is further evidence of abnormal properties of the cerebellar motor network in CD.

**Supplementary Information:**

The online version contains supplementary material available at 10.1007/s00415-025-13186-x.

## Introduction

Dystonia is a movement disorder characterized by sustained or intermittent muscle contractions leading to abnormal postures, movements, or both [1]. Cervical dystonia (CD) is the most common form of focal isolated dystonia characterized by abnormal postures or movements of the head [2]. It has been considered a basal ganglia disorder for years [3, 4], but recent evidence of involvement of other brain regions has led to the proposal of a network model of dystonia [5]. The cerebellum appears to be important within the network model, but its pathophysiological role in dystonia remains unclear [6–8]. Reciprocal connections to other major nodes place it in a key position [9] within the sensorimotor network of the brain: the dentato-rubro-thalamic tract (DRTT) links it to the thalamus and the primary motor cortex (M1) [10] and animal studies showed mono- and disynaptic connections to the basal ganglia [11–13].

Several lines of evidence implicate the cerebellum in the pathophysiology of CD. Transcranial magnetic stimulation (TMS) revealed that the inhibitory influence of the cerebellum on M1 via cerebellar brain inhibition is impaired in CD; this inhibition is considered to be mediated by the cerebello-thalamo-cortical tract [14, 15]. In a study of acquired CD, half of the causal lesions identified on magnetic resonance images (MRI) were located within the cerebellum or the cerebellar peduncles and all lesions were functionally connected to the cerebellum [8]. The picture is less clear with the MRI studies of microstructural changes of cerebellar gray matter, where only a few studies found significant alterations in CD, and these revealed them in different locations within the cerebellum [16–18]. Structural connectivity of the DRTT, as assessed with diffusion-weighted imaging (DWI) and quantified with fractional anisotropy (FA), has been reported to be reduced in CD [19] and genetic dystonias [20, 21].

In a recent study, we investigated the influence of cerebellar transcranial direct current stimulation (ctDCS) on sensorimotor associative plasticity in isolated CD patients [22]. Sensorimotor plasticity was evoked by the TMS technique of paired associative stimulation (PAS) [23]. In this paradigm, electrical stimuli to the median nerve precede TMS pulses over the contralateral M1 repetitively at low frequency. Typically, PAS leads to a long-lasting increase in M1 excitability [24]. Both our study and a previous study by Hamada and colleagues [25] showed that anodal and cathodal ctDCS disrupted the PAS effect in healthy controls (HC), which suggests that the protocol induces plasticity via a transcerebellar pathway. In contrast, cathodal ctDCS failed to modulate PAS in CD patients. This finding is further evidence that the cerebellum plays a role in the pathophysiology of isolated CD. Still, the mechanisms underlying the different ctDCS effects in HC and CD remain unclear.

Non-invasive brain stimulation (NIBS) paradigms are characterized by large inter- and intra-individual variability [26–28]. This issue also pertains to ctDCS, in particular [29, 30]. Determinants of this variability may play a key role in translating NIBS to clinical applications to modulate pathological motor network alterations or behavior. Studies that link functional and structural properties of the cerebellar motor network may provide new insights both in health and disease.

In the current study, we first assessed the structural connectivity of the DRTT using DWI-MRI and individual probabilistic tractography in isolated CD patients and HC and then investigated the association between the structural connectivity and the effect that ctDCS had on PAS. Our aim was to explore whether the individual microstructural state of efferent cerebellar fibers was associated with the influence of ctDCS in HC and CD.

## Methods

### Participants

Eighteen patients with isolated CD were recruited in the outpatient clinic of the Department of Neurology of the University Medical Center Hamburg-Eppendorf. All patients received regular treatment with botulinum toxin injections and were investigated 9–12 weeks after the last injection. A group of HC subjects matched by age, sex, and handedness was included in this study for comparison. None of the patients and HC had other neurological disorders or were taking any centrally-acting medication. Patients with severe head tremor were excluded.

Before entering the study, all participants underwent a neurological examination. In CD patients, the severity of motor symptoms was evaluated using the motor subscore of the Toronto Western Spasmodic Torticollis Rating Scale (TWSTRS) [31]. The clinical examination was recorded on video for blinded rating by an experienced movement disorder specialist (SZ). Demographic data, such as age, sex, and disease duration in patients, were collected and handedness was assessed using the Edinburgh Handedness Inventory [32].

### Brain imaging

MRI data were acquired on a 3 T Siemens Magnetom Prisma scanner (Siemens Healthineers, Forchheim, Germany) with a 64-channel head coil. High-resolution T1-weighted (Magnetization Prepared Rapid Acquisition Gradient Echo, MPRAGE) and susceptibility-weighted (SWI) structural images and DWI images were acquired with the following parameters. MPRAGE: echo time (TE) = 2.15 ms, repetition time (TR) = 2500 ms, inversion time (TI) = 1100 ms, flip angle = 8°, voxel resolution = 0.8 × 0.8 × 0.8 mm. SWI: TE = 20 ms, TR = 27 ms, flip angle = 15°, voxel resolution = 0.8 × 0.8 × 1.2 mm. DWI: TE = 76 ms, TR = 6800 ms, voxel resolution = 1.8 × 1.8 × 1.8 mm, gradients *b* = 0, 500, 1000, and 2000 s/mm^2^, 96 non-collinear gradient directions.

#### Image processing

Image processing was conducted using the FSL (v6.0.4, http://www.fmrib.ox.ac.uk/fsl) and MRtrix (v. 3.0.2, https://www.mrtrix.org) software packages. The raw DWI data underwent denoising (MRtrix’ command *dwidenoise*), removal of Gibbs ringing artifacts (*mrdegibbs*), correction of motion, susceptibility, and eddy current artifacts (*dwifslpreproc*) and bias field correction (*dwibiascorrect*). Brain extraction was conducted with FSL’s brain extraction tool *bet*.

#### Segmentation of the regions of interest

The dentate nucleus (DN) and the thalamus were segmented to acquire the necessary regions of interest (ROI) for probabilistic tractography of the DRTT. An experienced MRI investigator (KG) performed the DN segmentation manually. The DN was identified on the SWI images by its characteristic shape that resembles a sac with lobed walls and segmented using the segmentation tool ITK-SNAP [33]. The thalamus was segmented automatically with FSL’s FIRST tool on the basis of the T1-weighted images. Finally, both ROIs were registered to the subject’s diffusion space using linear registration with 12 degrees of freedom provided by FSL’s FLIRT tool. Both thalamus segmentation results and registration fits were visually verified.

#### Individual probabilistic tractography and estimation of tract-related white matter integrity

Fiber-orientation distributions (FOD) were acquired with multi-shell multi-tissue constrained spherical deconvolution. Tractography of the DRTT was carried out with MRtrix’ Second-Order Integration over Fiber Orientation Distributions (iFOD2) probabilistic fiber-tracking algorithm using default specifications. 50,000 streamlines were created for each subject and side with the DN ROI as the seed, the thalamus ROI as the target, and an exclusion mask to prevent unplausible pathways. The generated streamlines were transformed into a track density image (TDI) and visually inspected for plausibility.

FA maps were calculated by fitting the diffusion tensor model at each voxel. Tract-related FA values for the DRTT were estimated at the superior cerebellar peduncles (SCP) within a cuboid extending from *x* = − 13 to 13, *y* = − 31 to − 46, and *z* = − 13 to − 38 in MNI space, similar to a previous study by Guder and colleagues [34]. Concretely, the TDI tract image was thresholded at 10% intensity, binarized, and multiplied with the cuboid boundary mask which had been registered to the subject’s diffusion space. The average FA values of the resulting SCP ROI were calculated from the FA maps and the FA values for the left and right SCP were averaged for each subject.

### Neurophysiological investigation

A subset of 13 CD patients and 13 HC subjects was enrolled in a neurophysiological experiment to explore how ctDCS affects sensorimotor associative plasticity. These 26 participants are part of a larger cohort that we reported before [22]. The details of the experimental setup have been published previously and will only be described briefly. Each subject participated in three sessions that took place a week apart. In each session, participants underwent one of three types of ctDCS—anodal, cathodal, or sham—followed by the PAS protocol [23]. Motor-evoked potentials (MEP) were recorded from the right abductor pollicis brevis muscle at certain time intervals up to 60 min after PAS to investigate sensorimotor plasticity. Absolute MEP amplitudes recorded 0, 15, 30, and 60 min after PAS and tDCS were expressed as percentages of the average of the corresponding baseline series. The experiments followed a randomized, double-blind cross-over design in which each participant received all types of ctDCS across the three sessions. For further details, see Grimm et al. 2023 [22].

### Data analysis and statistics

All statistics were carried out using R v4.3.0 (R Foundation for Statistical Computing, Vienna, Austria, https://www.R-project.org/). The package *lmerTest* and *lmtest* were used for linear mixed modeling and likelihood ratio testing, respectively [35].

The comparison of FA values between groups was analyzed with a linear regression model with FA as the dependent, GROUP as the independent, and AGE as a nuisance variable. The relationship between motor symptom severity, as assessed with TWSTRS, and FA was modeled with TWSTRS as the dependent and FA and AGE as the independent variables. From the neurophysiological experiment, only data of from sham and cathodal ctDCS interventions were included in the statistical analysis, since only the cathodal ctDCS effect differed between HC and CD [22]. The effect of ctDCS on PAS was analyzed with a linear mixed model with the fixed-effects GROUP (two levels: CD and HC), TIME (four levels: 0/15/30/60 min after PAS), and INTERVENTION (two levels: cathodal and sham ctDCS), the random effect SUBJECT (one level for each subject), and an interaction of GROUP and INTERVENTION. Finally, the association between DRTT structure and ctCDS influence was investigated. To this end, we constructed a derivative ctDCS influence variable (ctDCS effect = PAS effect in cathodal ctDCS condition – PAS effect in sham condition). In a regression model, the ctDCS effect variable was treated as dependent with FA, GROUP, and AGE as independent variables. The model included an interaction between FA and GROUP. This association was then also tested separately for both groups and model validity was tested with likelihood ratio tests with and without FA as an independent variable.

Predictors and 95% confidence intervals are reported. For clarity, the average results of the four TIME levels after ctDCS and PAS are given in the results section. p values of < 0.05 were considered statistically significant. Due to the study’s exploratory nature, an adjustment for multiple tests was omitted.

## Results

### Subjects

Eighteen patients with isolated CD (10 females, median age 59 years [range 44–76]) and 18 HC (11 females, median age 58.5 years [42–78]) matched by age, sex, and handedness were included in this study. Of these, 13 subjects of both groups were enrolled in the neurophysiological investigation (CD: 6 females, median age 59 years [44–73]; HC: 7 females, median age 60 years [44–78]). Demographic and clinical data of the patients are shown in Table 1.

**Table 1:** Demographic and clinical data of the cervical dystonia patients

ID	Age	Sex	Duration	TWSTRS	Description of dystonic posture	TMS
CD01	58	M	15	4	Torticollis to the left	y
CD02	54	M	10	8	Torticollis to the right, retrocollis	y
CD03	44	F	9	21	Torticollis to the right, laterocollis to the right, shift to the left	y
CD04	73	M	20	21	Torticollis to the right, laterocollis to the left, anterior shift	y
CD05	60	M	20	21	Laterocollis to the left, shift to the right	y
CD06	61	F	2	20	Torticollis to the left, laterocollis to the right, shift to the right	y
CD07	45	F	2	15	Laterocollis to the right, retrocollis	y
CD08	50	F	11	8	Torticollis to the right, retrocollis	y
CD09	66	M	5	12	Torticollis to the right, laterocollis to the left	y
CD10	51	F	9	21	Torticollis to the right, laterocollis to the left, retrocollis	y
CD11	59	F	1	20	Torticollis to the left, laterocollis to the right, shift to the left	y
CD12	65	M	4	23	Torticollis to the right, laterocollis to the right, retrocollis	y
CD13	59	F	14	18	Torticollis to the left, laterocollis to the right, retrocollis	y
CD14	44	F	9	7	Torticollis to the right	n
CD15	54	F	10	12	Torticollis to the left	n
CD16	60	F	5	9	Torticollis to the right	n
CD17	76	M	8	17	Torticollis to the left, Laterocollis to the left, shift to anterior	n
CD18	66	M	8	22	Torticollis to the right, laterocollis to the left, retrocollis	n

Duration refers to the number of years the patients suffered from cervical dystonia. TMS refers to whether or not (n) the subject was included in the electrophysiological study. *TWSTRS* motor subscore of the Toronto Western Spasmodic Torticollis Rating Scale, *TMS* transcranial magnetic stimulation

### Comparison of DRTT FA across groups

**Fig. 1:**
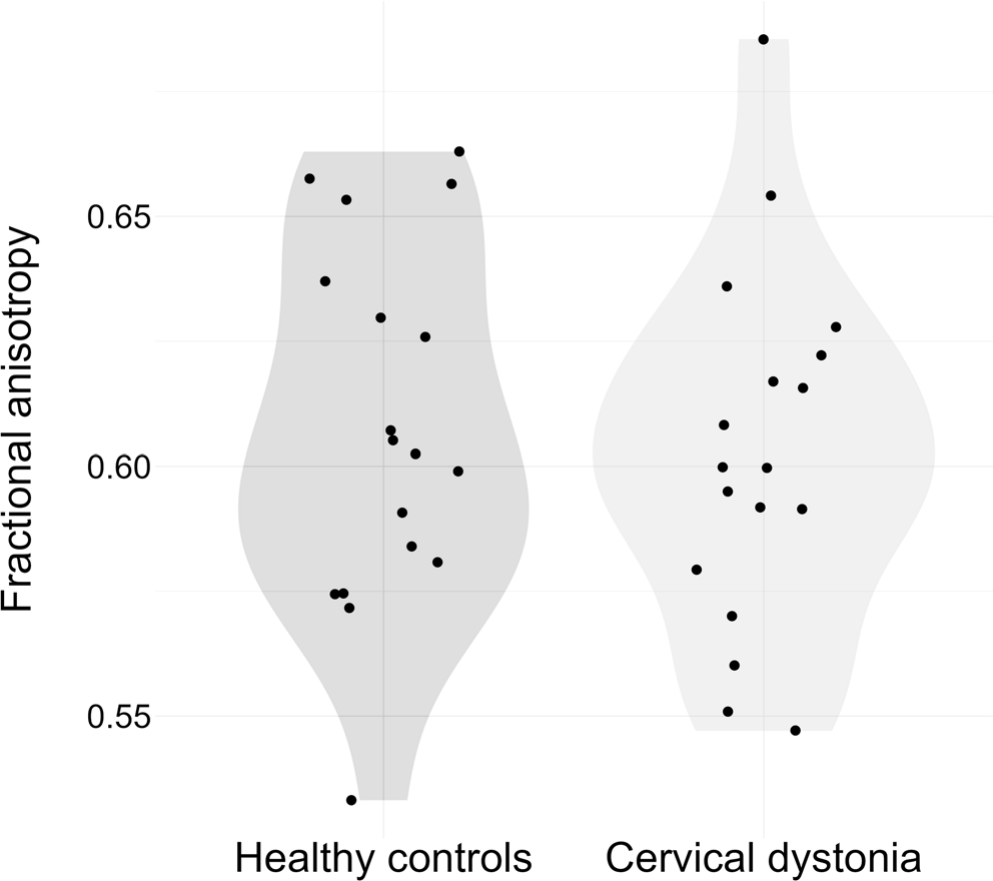
Violin plot and individual data points that show the structural connectivity of the DRTT in healthy control subjects and cervical dystonia patients. Structural connectivity is operationalized as the fractional anisotropy at the level of the superior cerebellar peduncle. There is no statistically significant group difference. DRTT: dentato-rubro-thalamic tract

The DRTT was successfully reconstructed in all subjects. As expected on the basis of previous studies [34, 36], visual inspection of TDI images showed that the streamlines exit the cerebellum via the ipsilateral SCP (see Online Resource 1), cross in the mesencephalon, and enter the thalamus ventrolaterally. The estimate for FA of the DRTT in the vicinity of the SCP was 0.603 [95% confidence interval: 0.587–0.619] in the CD and 0.608 [0.592–0.624] in the HC group (*p* = 0.633). The FA values did not differ between groups (see Fig. 1). Furthermore, there was no significant association of motor symptom severity assessed by the TWSTRS and FA of the DRTT (*p* = 0.74).

### Effect of ctDCS on PAS

**Fig. 2:**
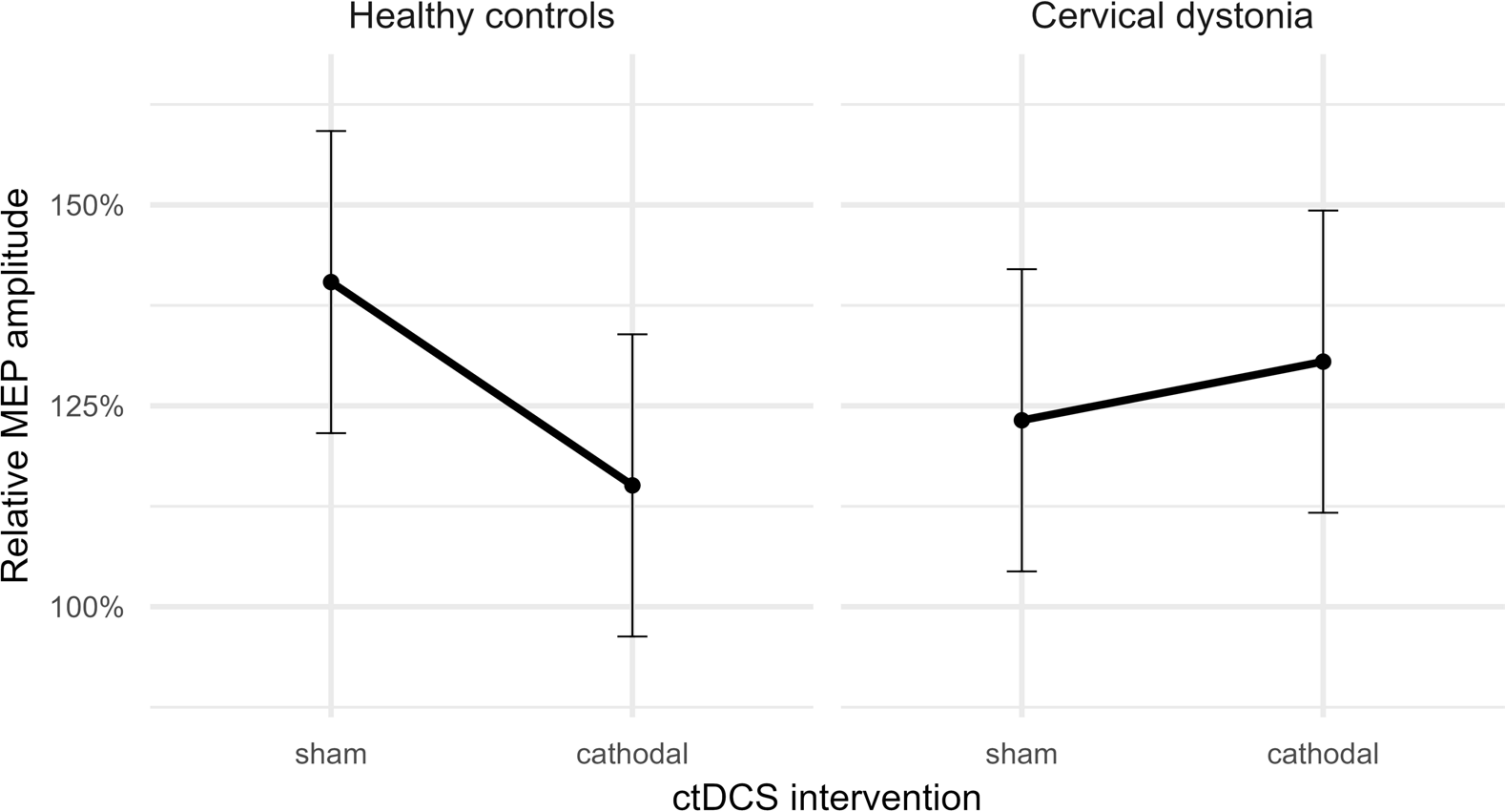
The PAS effect in both groups and in both experimental conditions (sham and cathodal ctDCS) is shown relative to the pre-interventional baseline. The ctDCS intervention reduced the PAS effect in the control group. There was no significant effect of ctDCS in the cervical dystonia group. Error bars represent the 95% confidence interval. MEP: motor-evoked potential, ctDCS: cerebellar transcranial direct current stimulation, PAS: paired associative stimulation

There was a significant effect of INTERVENTION (*p* = 0.003) but not GROUP (*p* = 0.993) in the neurophysiological investigation assessing the effect of cathodal ctDCS on PAS. The interaction between INTERVENTION and GROUP was significant (*p* < 0.001, see Fig. 2). Post hoc testing showed that the PAS effect was significantly attenuated by cathodal ctDCS in the HC group. Cathodal ctDCS reduced the PAS effect from 39.7% [17.7–61.6] to 15.2% [− 6.8–37.2] (*p* < 0.001). In contrast, in CD patients, PAS was not significantly different after cathodal and sham ctDCS (*p* = 0.072). PAS led to an increase in MEP amplitudes of 23.6% [1.7–45.6] in the sham tDCS condition and of 31.0% [9.0–52.9] after cathodal ctDCS.

### Association of DRTT structure and cathodal ctDCS effect

**Fig. 3:**
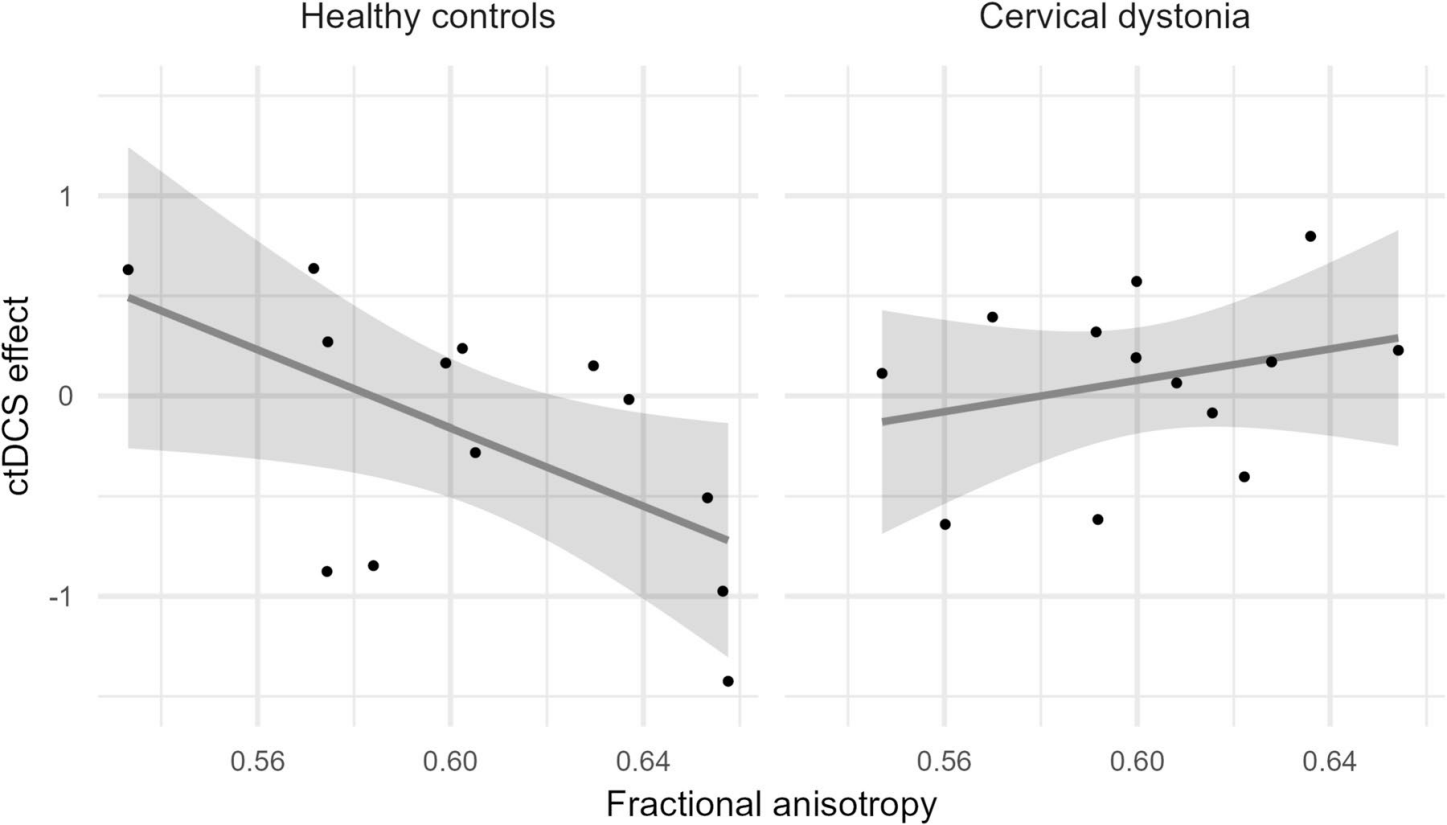
Association of the fractional anisotropy and the ctDCS effect in both groups. The individual data points are depicted alongside a linear regression line and 95% confidence intervals. The ctDCS effect is calculated as follows: Mean MEP amplitude after ctDCS and PAS in the verum condition minus mean MEP amplitude after the intervention in the sham condition. ctDCS: cathodal transcranial direct current stimulation, PAS: paired associative stimulation, MEP: motor-evoked potential

The linear regression model for the cathodal ctDCS effect on PAS showed a significant interaction of FA and GROUP (*p* = 0.017). This interaction was characterized by a negative slope in the HC group and a positive slope in the CD group, as illustrated by Fig. 3. FA was negatively associated with the cathodal ctDCS effect in the HC group (*p* = 0.039), i.e., higher FA of the DRTT was associated with a stronger inhibitory effect of cathodal ctDCS. In CD, however, there was no association between FA and the cathodal ctDCS effect on PAS (*p* = 0.486). The goodness of fit of the linear model for the HC group was significantly improved with the inclusion of FA (*p* = 0.016).

## Discussion

In this study, we assessed the structural connectivity of the DRTT, operationalized as FA, in CD patients and HC subjects. The results were compared across groups and the association of DRTT connectivity and ctDCS effects on sensorimotor associative plasticity were investigated. There were two main findings: First, structural connectivity was not different between groups. Second, the individual FA values of the DRTT were associated with the influence of cathodal ctDCS in HC but not in CD patients.

### Structural connectivity of the DRTT did not differ between groups

This is the first study to assess the microstructural state of the DRTT using individual probabilistic tractography in isolated CD patients. No group difference was found for the FA values corresponding to the part of the DRTT at the level of the SCP. Furthermore, there was no association of FA values with motor symptom severity in the CD group.

There is one other group that investigated the structural connectivity of the DRTT in idiopathic CD patients [19]. Sondergaard and colleagues reported that FA values of the right DRTT were 6.1% higher in the HC group than in the CD group, while those of the left DRTT did not differ. There are several important differences between the studies that restrict direct comparability. Most importantly, in the current study, FA values were considered in a section of the DRTT that corresponded to the SCP using individual probabilistic tractography. This approach increases the likelihood that the resulting FA values reflect the target tract. In contrast, Sondergaard and colleagues applied a DRTT template generated from a group of HC to FA maps of CD patients and extracted FA values from the entire tract mask. Evaluating the entire DRTT in this way may capture meaningful group differences that our approach did not detect, but due to general methodological limitations of probabilistic tractography, these differences may also be explained by adjacent structures other than the target tract, e.g., cerebral spinal fluid. Also, the patient cohorts of the two studies differed. In the present study, only isolated CD patients were included. In contrast, a third of the patients in the study by Sondergaard and colleagues also had dystonia in other body regions. Similar to our results, no association between DRTT connectivity and motor symptom severity was reported in the previous study [19].

### Structural connectivity is associated with ctDCS effect only in HC subjects

The other main finding was that the FA of the DRTT was related to the effect of cathodal ctDCS on PAS in HC subjects. Higher connectivity of the DRTT was associated with a greater effect of cathodal ctDCS. Thus, the FA of the DRTT may be a suitable biomarker for the efficacy of ctDCS in HC.

There is evidence that ctDCS can modulate cerebellar motor and non-motor functions [37, 38]. However, the effects of tDCS, in general, and ctDCS, in particular, are subject to both inter-subject and inter-study variability, which impedes its application in both research and clinical practice [29, 30, 39]. For this reason, biomarkers that can predict the effectiveness of tDCS are highly desirable [40]. An emerging line of evidence suggests that the effects of NIBS, i.e., tDCS and TMS, may be associated with the structural and functional connectivity within relevant brain networks of the investigated cohorts [41–45]. The present study represents the first piece of evidence that links the connectivity within the cerebellar motor network to the effectiveness of ctDCS. Further studies are necessary to validate these findings and investigate this association with regard to other physiological and behavioral effects of ctDCS.

Interestingly, the association between DRTT connectivity and ctDCS effects could not be found in CD patients. One possible explanation is that that the structure–function association of the cerebellar motor network is pathologically decoupled in isolated CD. Following this interpretation, the present findings may support the notion that cerebellar sensorimotor processing is impaired in CD and that not only the cerebellum per se is involved, as suggested by Hamada and colleagues [25], but also that the efferent cerebellar outflow projections are engaged in the network pathology. However, it is also possible that this result merely reflects reduced responsiveness to ctDCS in CD patients.

### Limitations

There are several limitations to this study. First, the sample size is small which is a common limitation in neurophysiological and imaging studies of CD [46]. Second, our insufficient understanding of the underlying cellular mechanisms of both PAS and ctDCS limits the interpretation of the current findings. Third, FA reflects the restriction of water molecule diffusion along the axis of the applied gradient, averaged over the voxel [47]. Mapping this phenomenon to a higher-level concept, i.e., connectivity, relies on implicit assumptions that need to be considered. Furthermore, the fibers reconstructed by probabilistic tractography and represented by FA values are much smaller than the resolution of the MR image, i.e., the size of a given voxel. Therefore, the resulting value may reflect properties of neighboring microstructures as well as those of the target tract. That being said, performing tractography and using FA to investigate white matter connectivity are common and justifiable as long as these limitations are kept in mind. Finally, it cannot be ruled out entirely that long-term effects of botulinum toxin on the central nervous systems influenced our neurophysiological measures [48].

## Conclusion

The present results suggest that the individual structural connectivity within the cerebellar motor network may serve as a biomarker for the responsiveness to cathodal ctDCS in HC subjects. Since this structure–function association is impaired in CD patients, the microstructural state of the DRTT is not able to predict ctDCS effects on PAS in CD. This is further evidence of cerebellar motor network pathology in CD.

## Supplementary Information

Below is the link to the electronic supplementary material.

Supplementary file1 (PDF 2260 KB)

## Data Availability

The data that support the findings of this study are available from the corresponding author, SZ, upon reasonable request.
