# Supplementary material for: Structure–function association of the cerebellar motor network is altered in isolated cervical dystonia
Source: J Neurol. 2025 Jun 3;272(6):441. doi: 10.1007/s00415-025-13186-x (PMC12133984; doi:10.1007/s00415-025-13186-x)
Supplement: Supplementary file 1 — Supplementary file1 (PDF 2260 KB) [file 415_2025_13186_MOESM1_ESM.pdf]

Journal of Neurology

Structure-function association of the cerebellar motor network is altered in isolated cervical dystonia

Kai Grimm, MD[1]; Hanna Braaß, MD[1,2]; Fatemeh Sadeghi, MSc[1]; Mathias Gelderblom, MD[1]; Robert Schulz, MD[1]; Simone Zittel, MD[1]

[1] Department of Neurology, University Medical Center Hamburg-Eppendorf, Hamburg, Germany
[2] Institute of Systems Neuroscience, University Medical Center Hamburg-Eppendorf, Hamburg, Germany

Corresponding author:
Dr. Simone Zittel
s.zittel-dirks@uke.de

Electronic supplementary material 1. Results of probabilistic tractography of the DRTT in cervical dystonia patients and healthy control subjects. The left images show axial and the right images show coronal slices of the colored tracts superimposed onto the FA maps at the level of the SCP. Tracts originating from the right dentate nucleus are represented in blue and those originating from the left dentate nucleus in red. The color intensity reflects the streamline density as represented by the tract density images. The reference point (max) is the voxel within the given tractogram that is crossed by the highest number of reconstructed streamlines. The color scale represents percentages of that maximum. The maximum itself is the upper limit and 10% of that maximum value is the lower limit. DRTT: Dentato-rubro-thalamic tract, FA: fractional anisotropy, SCP: superior cerebellar peduncle.
Supplementary Figure created with GNU Image Manipulation Program.

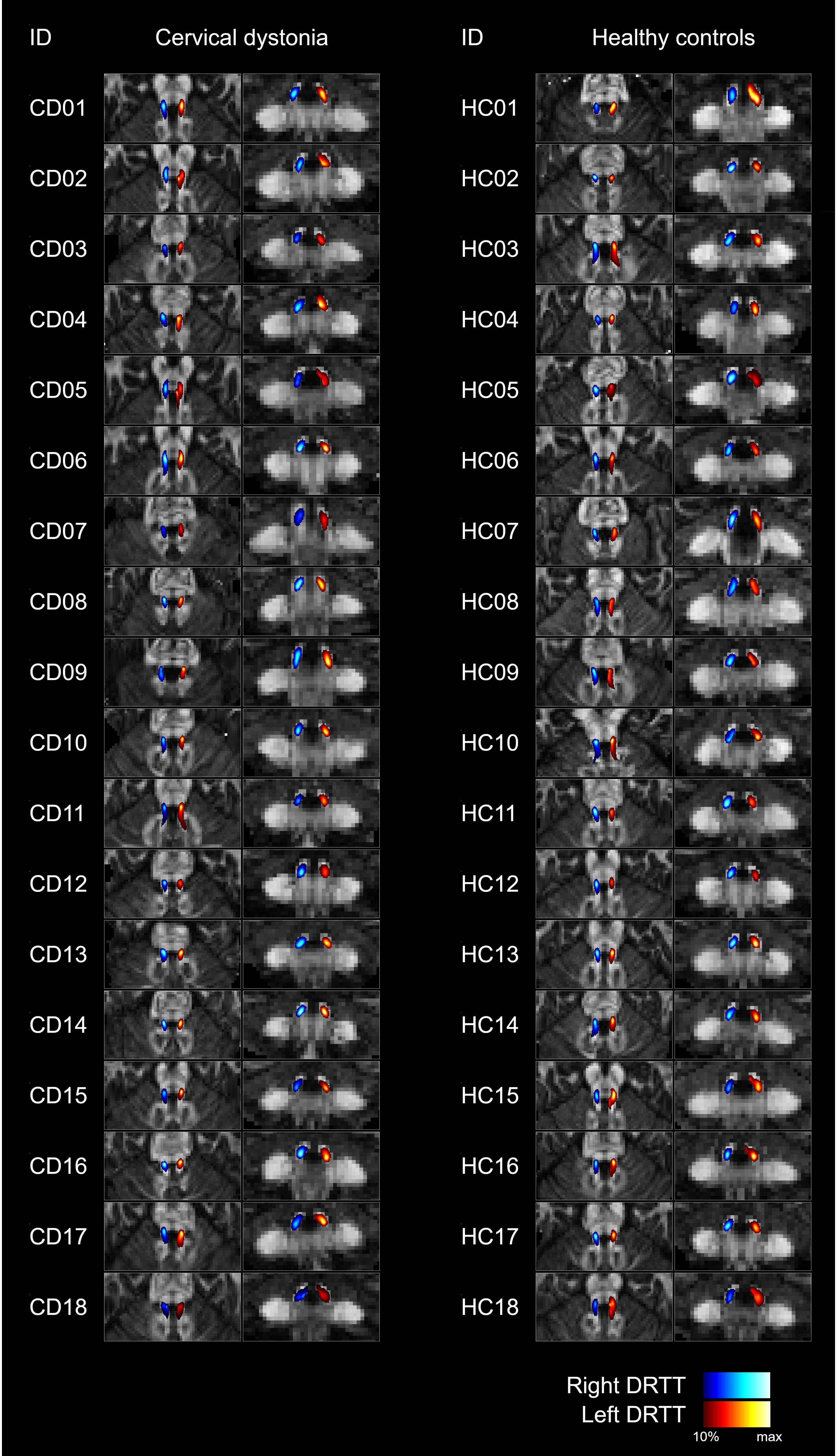
ID
Cervical dystonia
ID
Healthy controls
CD01
CD02
CD03
CD04
CD05
CD06
CD07
CD08
CD09
CD10
CD11
CD12
CD13
CD14
CD15
CD16
CD17
CD18
HC01
HC02
HC03
HC04
HC05
HC06
HC07
HC08
HC09
HC10
HC11
HC12
HC13
HC14
HC15
HC16
HC17
HC18
Right DRTT
Left DRTT
10%
max
